# Supplementary figures and images for: Next-Generation Sequencing for Venomics: Application of Multi-Enzymatic Limited Digestion for Inventorying the Snake Venom Arsenal
Source: Toxins (Basel). 2023 May 25;15(6):357. doi: 10.3390/toxins15060357 (PMC10304959; doi:10.3390/toxins15060357)

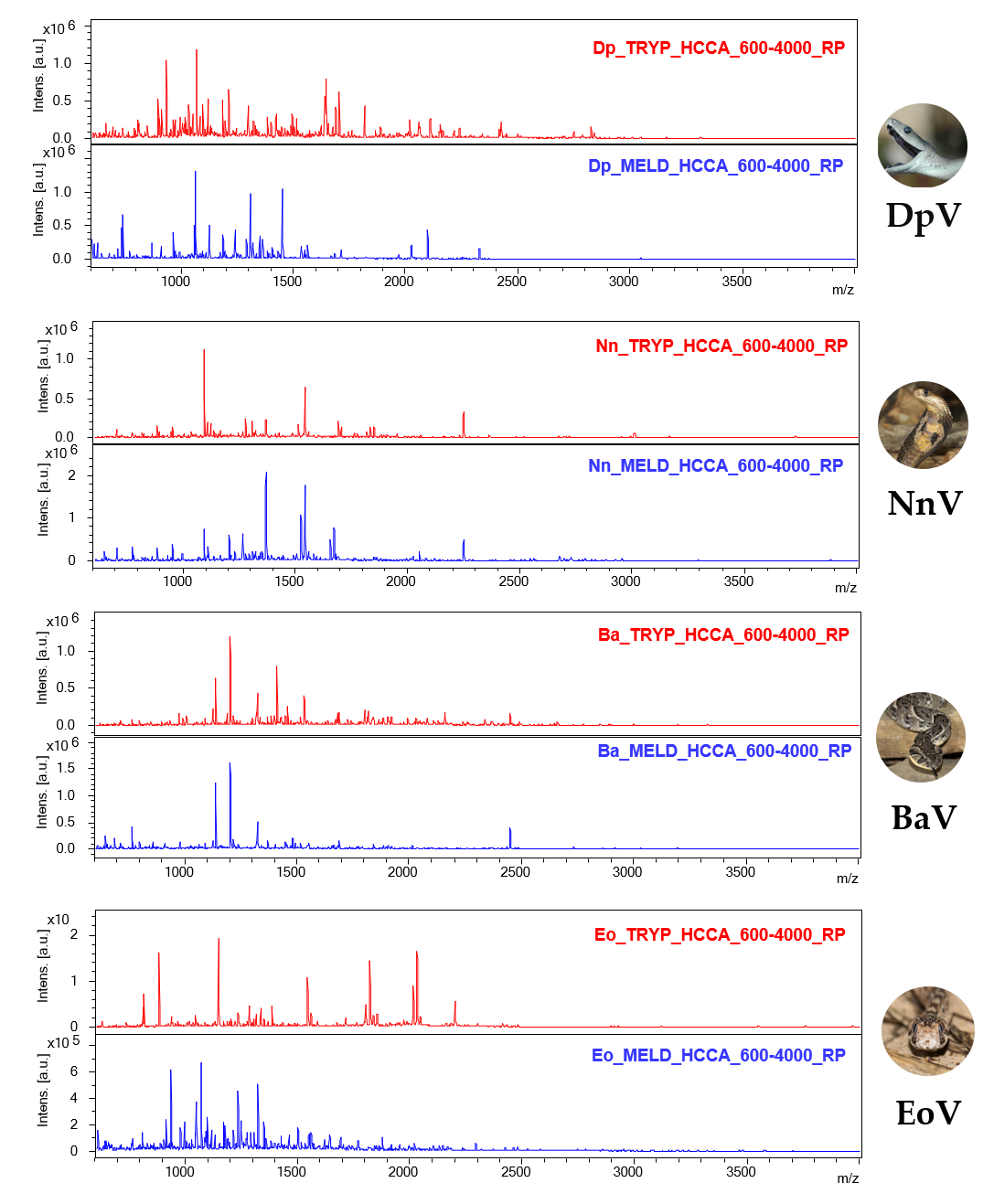

Supplement: Supplementary file 1 [file toxins-15-00357-s001.zip › toxins-2356921-Supplementary File S1/S1_supplementary material.tif]
